# Image analysis with deep learning to predict breast cancer grade, ER status, histologic subtype, and intrinsic subtype

**DOI:** 10.1038/s41523-018-0079-1

**Published:** 2018-09-03

**Authors:** Heather D. Couture, Lindsay A. Williams, Joseph Geradts, Sarah J. Nyante, Ebonee N. Butler, J. S. Marron, Charles M. Perou, Melissa A. Troester, Marc Niethammer

**Affiliations:** 1 Department of Computer Science, University of North Carolina at Chapel Hill, Chapel Hill, NC 27599 USA; 2 Department of Epidemiology, University of North Carolina at Chapel Hill, Chapel Hill, NC 27599 USA; 3 Department of Pathology, Dana-Farber Cancer Institute, Boston, MA 02115 USA; 4 Department of Radiology, University of North Carolina at Chapel Hill, Chapel Hill, NC 27599 USA; 5 Lineberger Comprehensive Cancer Center, University of North Carolina at Chapel Hill, Chapel Hill, NC 27599 USA; 6 Department of Statistics and Operations Research, University of North Carolina at Chapel Hill, Chapel Hill, NC 27599 USA; 7 Department of Genetics, University of North Carolina at Chapel Hill, Chapel Hill, NC 27599 USA; 8 Biomedical Research Imaging Center, University of North Carolina at Chapel Hill, Chapel Hill, NC 27599 USA

## Abstract

RNA-based, multi-gene molecular assays are available and widely used for patients with ER-positive/HER2-negative breast cancers. However, RNA-based genomic tests can be costly and are not available in many countries. Methods for inferring molecular subtype from histologic images may identify patients most likely to benefit from further genomic testing. To identify patients who could benefit from molecular testing based on H&E stained histologic images, we developed an image analysis approach using deep learning. A training set of 571 breast tumors was used to create image-based classifiers for tumor grade, ER status, PAM50 intrinsic subtype, histologic subtype, and risk of recurrence score (ROR-PT). The resulting classifiers were applied to an independent test set (*n* = 288), and accuracy, sensitivity, and specificity of each was assessed on the test set. Histologic image analysis with deep learning distinguished low-intermediate vs. high tumor grade (82% accuracy), ER status (84% accuracy), Basal-like vs. non-Basal-like (77% accuracy), Ductal vs. Lobular (94% accuracy), and high vs. low-medium ROR-PT score (75% accuracy). Sampling considerations in the training set minimized bias in the test set. Incorrect classification of ER status was significantly more common for Luminal B tumors. These data provide proof of principle that molecular marker status, including a critical clinical biomarker (i.e., ER status), can be predicted with accuracy >75% based on H&E features. Image-based methods could be promising for identifying patients with a greater need for further genomic testing, or in place of classically scored variables typically accomplished using human-based scoring.

## Diagnostics: Image-based AI predicts breast cancer features

Artificial intelligence can accurately predict the histological subtype and molecular marker status of breast tumors from pathology slide pictures. Heather Couture from the University of North Carolina at Chapel Hill and coworkers developed a deep learning algorithm to analyze various molecular features found within pathology slide images from breast cancer tissue samples. They trained the algorithm on a set of 571 tumors with defined grade, subtype and hormone receptor status. They then tested the model on another set of 288 breast tumors, finding that the deep-learning method predicted assorted molecular features with an accuracy exceeding 75%. Artificial intelligence applied to breast tumor images in this way could thus help clinicians identify those patients who stand to benefit most from further RNA-based genomic testing, an expensive but more definitive diagnostic tool for disease classification.

## Introduction

Image-based features of breast cancers have an important role in clinical prognostics. For example, tumor grade is strongly associated with survivorship, even among tumors with other favorable prognostic features such as estrogen receptor positivity.^1^ However, major advances in prognostication over the past decade have relied predominantly on molecular methods.^2–4^ These methods are costly and are not routinely performed on all clinical patients who could benefit from advanced molecular tests. Methods for identifying patients who are likely to benefit from further molecular testing are needed.

Image analysis of hematoxylin and eosin (H&E)-stained images could identify patients most likely to benefit from genomic testing. Several previous studies have utilized automated processing of H&E stained breast tumors to identify image features associated with survival. These approaches have largely focused on hand-crafted, user-designed features, such as statistics of shape and color, to capture cell-by cell morphology, which are difficult to adapt to new data sets.^5,6^ Prior work on automated grading addresses mitotic count,^7^ nuclear atypia,^8^ and tubule formation^9^ individually; however, the latter two require a time-consuming nuclear segmentation that is also difficult to adapt to new data sets. Feature learning on small image patches to identify novel features associated with survival has shown the utility of somewhat more complex features for breast^10^ and other cancers,^11,12^ but the focus of that work still remains on smaller-scale properties due to their use of small image patches. None of these approaches is able to capture larger scale features, such as tissue architecture, or properties that are too complex for humans to capture. These abstract features could provide unforeseen insights into prognostics.

Deep learning is a method of learning a hierarchy of features where the higher level concepts are built on the lower level ones. Automatically learning these abstract features enables the system to learn complex functions mapping an input to an output without the need for hand-crafted features. Significant advances in this area have begun to show promise for tumor detection,^13^ metastatic cancer detection in lymph nodes,^14^ mitosis detection,^7,15^ tissue segmentation,^16^ and segmentation and detection of a number of tissue structures.^17^ However, all of the previous successes of deep learning from H&Es have focused on detecting image-based properties that pathologists can routinely assess visually. Using deep learning to predict complex properties that are not visually apparent to pathologists, such as receptor status, intrinsic subtype or even risk of recurrence, has not been previously described.

We hypothesized that a deep learning method for image analysis could be applied to classify H&E stained breast tumor tissue microarray (TMA) images with respect to histologic and molecular features. We used TMA images from the population-based Carolina Breast Cancer Study Phase 3 (2008–2013) to perform deep learning-based image analysis aimed at capturing larger scale and more complex properties including tumor grade, histologic subtype, estrogen receptor (ER) status, intrinsic breast cancer subtype, and Risk of Recurrence (ROR)-PT score.^2^

## Results

**Fig. 1:**
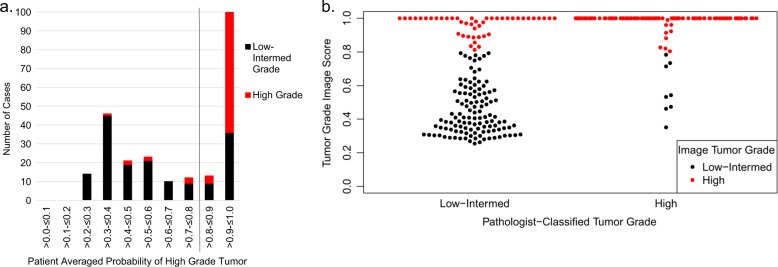
**a.** Histogram for probability of high-grade tumor by image analysis according to proportion of pathologist-classified low-intermediate (black) or high grade (red) in the test set. The cut point of >0.80 was selected. **b**. Bee Swarm plot displaying pathologist classification of tumor grade as a function of the image grade score in the test set. Points within each grade group are adjusted horizontally to avoid overlap. The black dots indicate image analysis classified low-intermediate tumor grade and the red dots indicate image analysis classified high-grade tumors

Training and test sets were established from a random division of the data using TMA cores from 2/3 (*n* = 571) and 1/3 (*n* = 288) of the eligible CBCS3 patients, respectively. There were no significant differences between the training and the test sets concerning patient or tumor characteristics (Table 1). Across multiple 1.0-mm cores per patient, the probability of a tumor being classified as high grade by image analysis was calculated, and Fig. 1 shows that a bimodal distribution of probabilities was observed. By establishing a cut point at > 0.80, high-grade tumors were detected with accuracy of 82% in the test set (*kappa* 0.64) (Figure 1a, b and Table 2). Considering low/intermediate as a group, the percent agreement with pathologist-classified tumor grade was slightly lower than the percent agreement between two breast pathologists who independently reviewed these same patients (overall 89%, *kappa* 0.78). Tumors with pathologist-defined intermediate grade were more likely to be misclassified as high-grade tumors by image analysis (37%), while only 7% of low-grade tumors were misclassified (results not shown). When comparing the misclassification of intermediate grade and low-grade tumors as high grade between two pathologists in a subset of CBCS tumors, errors in classification of intermediate grade tumors as high-grade tumors occurred <10% of the time and never occurred for low-grade tumors (results not shown).

**Table 1:** Patient and tumor characteristics for the image analysis training and test set, CBCS3

	Training set (*N* = 571) *N* (%^a^)	Test set (*N* = 288) *N* (%^a^)	Chi-square *p*-value
Age			
≤50 years	280 (29.6)	133 (28.0)	0.64
>50 years	291 (70.4)	155 (72.0)	
Race			
White	298 (79.0)	150 (78.7)	0.90
African-American	272 (21.0)	138 (21.3)	
Missing	1		
Grade			
Low-intermediate	330 (65.8)	162 (66.5)	0.85
High	240 (34.2)	125 (33.5)	
Missing	1	1	
Stage			
I, II	485 (86.4)	259 (90.2)	0.17
III, IV	85 (13.6)	29 (9.8)	
Missing	1		
Node status			
Negative	354 (65.2)	191 (69.1)	0.35
Positive	214 (34.8)	97 (30.9)	
Missing	3		
Tumor size			
≤2 cm	334 (62.5)	174 (67.2)	0.26
>2 cm	235 (37.5)	114 (32.8)	
Missing	2		
ER status			
Negative	164 (24.9)	91 (23.1)	0.62
Positive	405 (75.1)	197 (76.9)	
Missing	2		
PAM50 subtype			
Luminal A	149 (46.1)	74 (47.1)	0.27
Luminal B	78 (18.2)	33 (20.9)	
Basal-like	92 (20.9)	49 (21.6)	
HER2	46 (11.9)	15 (5.9)	
Normal-like	9 (2.9)	9 (4.5)	
Missing	197	108	

^a^All percentages weighted for sampling design

**Table 2:** Agreement between pathologists and between pathologists and image analysis in the test set for low-intermediate grade and high-grade tumors, CBCS3

Pathologist agreement on tumor grade classification^a^ (*n* = 242)			Image analysis agreement with pathologist tumor grade classification^b^ (*n* = 288)		
	Pathologist 2			Clinical grade	
Pathologist 1	Low-intermediate grade	High grade	Patient average grade	Low-intermediate grade	High grade
Low-intermediate grade	113	23	Low-intermediate grade	118	8
High grade	4	102	High grade	45	117
	% Agreement	89	% Agreement	82	
	*kappa* (95% CI)	0.78 (0.70–0.86)	*kappa* (95% CI)	0.64 (0.55-0.72)	

^a^To assess agreement between two pathologists, patients were sampled from CBCS Phases 1, 2, and 3 for second pathology review

^b^To assess agreement between image analysis and a pathologist, only samples with digital image data (CBCS3 only) were included

Image analysis accuracy for predicting molecular characteristics was also high. Accuracy for ER status was 84% (*kappa* 0.64*)* and both sensitivity (88%) and specificity (76%) were high (Table 3). However, tumor grade is strongly associated with ER status in most patient populations, and we were interested in increasing accuracy among patients with low-to-intermediate grade tumors where genomic testing is most likely to influence patient care. Thus, we also employed a training strategy that weighted samples to ensure that low and intermediate grade distributions were similar between ER-positive and ER-negative tumors. This reduced accuracy among high-grade tumors (from 77 to 75%), and decreased accuracy among low-intermediate grade tumors (from 91 to 84% accuracy). Using the same weighting strategy, we trained a classifier to predict Basal-like vs. non-Basal-like (Luminal A, Luminal B, HER2, Normal-like combined) PAM50 subtype (Table 4). The classifier had overall accuracy of 77%, but accuracy of 85% among low-intermediate grade tumors and 70% among high-grade tumors.

**Table 3:** Impact of weighting by grade on accuracy, sensitivity, and specificity of ER status^1^ in the test set, CBCS3

	Unweighted						Grade-trained					
	IHC ER status						IHC ER status					
Image analysis	Negative	Positive	Sensitivity (%)	Specificity (%)	Accuracy (%)	*Kappa* (95% CI)	Negative	Positive	Sensitivity (%)	Specificity (%)	Accuracy (%)	*Kappa* (95% CI)
Overall												
ER negative	260	80	88	76	84	0.64 (0.59–0.69)	246	104	84	72	80	0.55 (0.50–0.61)
ER positive	83	572					97	548				
Low-intermediate grade												
ER negative	21	24	95	46	91	0.41 (0.28–0.55)	28	69	86	61	84	0.31 (0.21–0.42)
ER positive	25	467					18	422				
High grade												
ER negative	239	46	69	80	77	0.49 (0.40-.57)	218	35	78	73	75	0.48 (0.44–0.56)
ER positive	58	104					79	125				

^a^Numbers represent individual cores (*n* = 995) from 288 patients, with up to four cores per patient; H&E cores were excluded if missing IHC data (*n* = 11)

**Table 4:** Accuracy, sensitivity, and specificity of non-Basal-like intrinsic subtype, ROR-PT, and histologic subtype based on image analysis^a^ in the test set, CBCS3

Image analysis	**Intrinsic subtype** ^**b**^					
	Basal-like	Non-Basal-like	Sensitivity (%)	Specificity (%)	Accuracy (%)	*Kappa* (95% CI)
Overall						
Basal-like	131	101	78	73	77	0.47 (0.32–0.54)
Non-Basal-like	48	368				
Low-intermediate grade						
Basal-like	11	41	86	73	85	0.27 (0.13–0.41)
Non-Basal-like	4	245				
High grade						
Basal-like	120	60	67	73	70	0.40 (0.31–0.50)
Non-Basal-like	44	123				
	**ROR-PT status** ^**b**^					
	Low-Med	High	Sensitivity (%)	Specificity (%)	Accuracy (%)	*Kappa* (95% CI)
Overall						
Low-Med	342	40	79	74	76	0.47 (0.40–0.54)
High	118	148				
Low-intermediate grade						
Low-med	245	16	47	90	86	0.32 (0.17–0.48)
High	26	14				
High grade						
Low-med	97	24	85	51	67	0.35 (0.26–0.44)
High	92	134				
	**Histologic subtype** ^**c**^					
	Ductal	Lobular	Sensitivity (%)	Specificity (%)	Accuracy (%)	*Kappa* (95% CI)
Overall						
Ductal	710	24	71	96	94	0.66 (0.57–0.74)
Lobular	28	58				
Low-intermediate grade						
Ductal	268	24	71	94	89	0.63 (0.53–0.73)
Lobular	23	58				
High grade						
Ductal	442	0	N/A	99	99	N/A
Lobular	5	0				

^a^Numbers represent individual cores from patients where 1–4 cores were available. Cores were excluded if RNA data (*n* = 358) was missing

^b^One-hundred eighty patients with 648 cores for intrinsic subtype and ROR-PT

^c^Two-hundred thirty-three patients with 820 cores for histologic subtype

To examine the potential clinical relevance of using this image analysis technique, we determined the sensitivity and specificity of image analysis and the ability to predict whether or not a tumor is classified as having high vs. low-medium risk of recurrence score (ROR-PT) (Table 4). ROR-PT is determined using a combination of tumor information including PAM50 subtype, tumor proliferation, and tumor size.^2^ Overall the accuracy of image analysis for ROR-PT was high at 76% (*kappa* 0.47*)*. In grade-stratified analyses, accuracy for ROR-PT was higher among low-intermediate grade tumors (86%) than high-grade tumors (67%).

In addition to using image analysis to predict tumor grade, we also tested this approach using histologic subtype, another visual feature of the tumor (Table 4). Image analysis was able to predict a lobular compared to ductal tumor with 94% accuracy (*kappa* 0.66). The accuracy was slightly lower when restricted to low-grade tumors (89%), but was non-estimable among high-grade tumors as there were no high-grade lobular tumors in the test set.

To evaluate which clinical factors were associated with the accuracy of the image-based metrics, we evaluated predictors of accurate/inaccurate ER status calls (Supplemental Table 1) among patients in the test set (*n* = 288). Considering age, race, grade, stage, lymph node status, ER status, Ki67 status, and mitotic tumor grade, no significant differences in accuracy of image-based ER assignment were observed. However, we found that image analysis tended to inaccurately predict ER status when tumors were Luminal B [OR, (95% CI); 4.42 (1.32–14.77)].

**Fig. 2:**
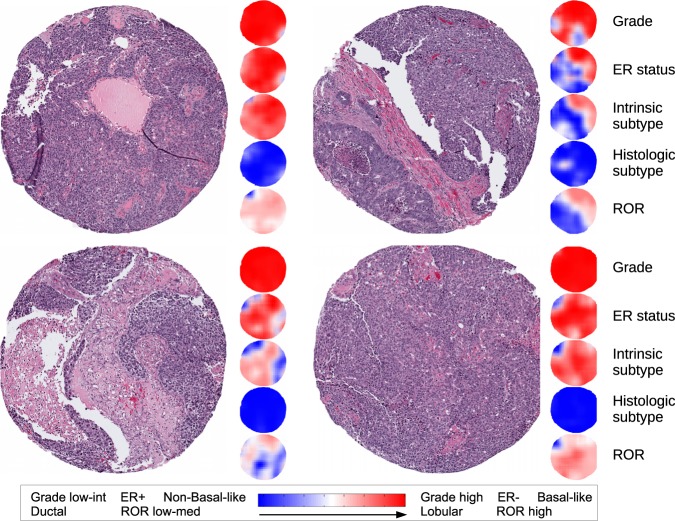
Four H&E cores from a single patient and heat maps indicating the class predictions over different regions of the image. Class probabilities are indicated by the intensity of red/blue color with greater intensity for higher probabilities. Uncertainty in the prediction is indicated by white. This patient was labeled as high grade, ER negative, Basal-like intrinsic subtype, ductal histologic subtype, and high ROR

We gained further insight into the performance of our method by examining the class predictions across cores from the same patient and within each core. Figure 2 shows four cores from a single patient, along with the class predictions over different regions of the image. While three cores are predicted ER negative and Basal-like intrinsic subtype, the fourth is predicted mostly ER negative and non-Basal-like, indicating that some intra-tumoral heterogeneity might be present between cores.

## Discussion

In this study, we used a deep learning approach to conduct image analysis on H&E stained breast tumor tissue microarray samples from the population-based Carolina Breast Cancer Study, Phase 3 (2008–2013). Further details on the image analysis techniques are given in the Methods section. First, we found that the agreement between image analysis and the pathologist-classified grade was only slightly lower than that observed for two study pathologists, and we obtained high agreement and *kappa* values. Second, we found that ER status, RNA-based molecular subtype (Basal-like vs. non-Basal-like), and risk of recurrence score (ROR-PT) could be predicted with approximately 75–80% accuracy. Further, we found the image analysis accuracy to be 94% for ductal vs. lobular histologic subtype.

Previous literature based on comparing two pathologists shows that image assessment is subject to some disagreement,^18^ particularly among the intermediate grade tumors as we observed between the image analysis and pathologist classification in our study. Other groups have reported inter-rater *kappa* statistics of 0.6–0.7 for tumor grade,^18,19^ in line with both our inter-pathologist agreement and image analysis vs. pathologist agreement for grade. Elsewhere in the literature lower *kappa* values around 0.5 have been reported between pathologists for histologic grade.^20^ In light of this inherent variability in image assessment, deep learning-based image analysis performed well at predicting tumor grade as low-intermediate vs. high using H&E images.

It is particularly promising that histologic subtype and molecular marker status could be predicted using image analysis. While we did perform grade-weighting within ER classification, there may be other image features of ER-positive tumors that are not readily discernible and are driving the higher accuracy of ER-positive images over ER negative. Agreement between true ER status (by immunohistochemistry (IHC)) vs. image analysis (*kappa* 0.64) was slightly lower than that observed for centralized pathology and SEER classifications for ER status (*kappa* 0.70)^21^ and is similar to reports of agreement between different IHC antibodies for ER that show substantial agreement (*kappa* 0.6–0.8).^22^ Previous work with CBCS phase 1 samples found that agreement between medical records and staining of tissues was also similar (*kappa* of 0.62).^23^ Overall, the agreement between IHC-based ER status and image analysis predictions based on H&E stained images are similar to estimates for comparing ER status classification in the literature. The high rate of agreement between pathologist-scored and image analysis based histologic subtype was also compelling (*kappa* 0.64). Altogether these results suggest that some latent features indicative of underlying tumor biology are present in H&E images and can be identified through deep learning-based approaches.

We observed high accuracy of image analysis to predict ductal versus lobular histologic subtype. The high accuracy may be due to the arrangement of epithelial and stromal cells characteristic of ductal and lobular tumors whereby lobular tumors are characterized by non-cohesive single file lines of epithelial cells infiltrating the stroma and ductal tumors are characterized by sheets or nests of epithelial cells embedded in the surrounding stroma.^24,25^ We speculate that it may be that the high contrast staining between the epithelium and stromal components resulting from H&E immunohistochemistry strengthens the ability of image analysis to predict this biologic feature of the tumor.

With respect to intrinsic PAM50 subtype based solely upon gene expression values, previous studies have not evaluated image-based analysis for predicting intrinsic subtype or the risk of recurrence using a score-based method, ROR-PT.^2^ A few previous studies have evaluated the clinical record or a central immunohistochemistry laboratory vs. RNA-based subtyping for Basal-like vs. non-Basal-like. Even considering two molecular comparisons, agreements do not exceed 90%. That is, Allott et al.^26^ found approximately 90% agreement between Basal-like status for IHC-based vs. RNA-based assessment and 77% agreement for classification of Luminal A subtype.^26^ Our estimates are similar suggesting that image analysis, even without the use of special IHC stains, could be a viable option for classification of molecular breast tumor subtype and ROR-PT from H&E stained images.

As with other studies, our work should be viewed in light of some limitations. Our sample size was limited in our testing set to 288 patients, but this resulted in nearly 1000 TMA cores available for use in our image analysis. Using a larger set of samples with data on RNA-based subtype to balance training for each predictor could be useful. For example, the fact that Luminal B patients had a higher error rate might suggest there are some features of Luminal B breast cancers that are distinct and image-detectable, and a larger sample size would be helpful in identifying these. Deep learning may be utilizing these features, but in our small sample set, we are unable to tune our data to specifically identify those features or to clarify what they are in intuitive language. Additionally, the use of binary classification systems for training our digital algorithms (i.e., Basal-like vs. non-Basal-like) does not allow us to differentiate among all five RNA-based intrinsic subtypes. Currently, U.S.-based genomic tests provide continuous risk scores, but also suggest relevant cut points that in essence make these assays almost a binary classification; thus, binary classification may have some utility in the current clinical context. However, future work should extend these approaches to multiclass classification. Furthermore, improved results may be obtained by fine-tuning the Convolutional Neural Network for breast cancer H&E image classification.

Image-based risk prediction has potential clinical value. Gene expression data on tumor tissue samples is not uniformly available for all patients and is costly to obtain in both a clinical and epidemiologic setting. These results suggest that tumor histology and molecular subtype along with the risk of recurrence (ROR-PT) can be predicted from H&E images alone in a high-throughput, objective, and accurate manner. These results could be used to identify patients who would benefit from further genomic testing. Furthermore, even ER testing is not routinely performed in countries with limited laboratory testing resources and predicting ER status by morphologic features may have utility for guiding endocrine therapy in low-resource settings.

## Methods

### Sample set

The training and test sets were both comprised of participants from the Carolina Breast Cancer Study (CBCS), Phase 3 (2008–2013). Methods for CBCS have been described elsewhere.^27^ Briefly, CBCS recruited participants from 44 of the 100 North Carolina counties using rapid case ascertainment via the North Carolina Central Cancer Registry. After giving informed consent, patients were enrolled under an Institutional Review Board protocol that maintains approval at the University of North Carolina. CBCS eligibility criteria included being female, a first diagnosis of invasive breast cancer, aged 20–74 years at diagnosis, and residence in specified counties. Patients provided written informed consent to access tumor tissue blocks/slides and medical records from treatment centers.

The training and test sets were formed by a random partition of the data. The total number of patients available for the training and test set from CBCS3 was 1203. These patients were divided into a group of 2/3 (*n* = 802) for the training set and 1/3 (401) for the test set. Of the 802 patients available for the training set, 571 had H&E images and biomarker data available for contribution to the training set. Of the 401 patients eligible for the test set, 288 had H&E images and biomarker data available. Patients in the final training and test sets had information for tumor grade and histologic subtype, determined via centralized breast pathologist review within CBCS, along with biomarker data for ER status, PAM50 intrinsic breast cancer subtype, and risk of recurrence (ROR-PT) where noted. The H&E images were taken from tissue microarrays constructed with 1–4 1 mm cores for each patient, resulting in 932 core images for the test set analysis presented here. ER status for each TMA core was determined using a digital algorithm as described by Allott et al.^28^ and was defined using a ≥ 10% positivity cut point for immunohistochemistry staining.

### Tumor tissue microarray construction

As has been described in detail by Allott et al., tumor tissue microarrays were constructed for CBCS3 participants with available paraffin-embedded tumor blocks.^26^ The CBCS study pathologist marked areas of invasive breast cancer within a tumor on H&E stained whole slide images. The marked areas were selected for coring and 1–4 tumor tissue cores per participant were used in the TMA construction at the Translational Pathology Laboratory at UNC. TMA slides were H&E stained and images were generated at 20x magnification. Cores with insufficient tumor cellularity were eliminated from the analysis.

### Molecular marker data

In CBCS3, Nanostring assays were carried out on a randomly sampled subset of available formalin fixed paraffin-embedded (FFPE) tumor tissue cores. RNA was isolated from 2, 1.0-mm cores from the same FFPE block using the Qiagen RNeasy FFPE kit (catalog # 73504). Nanostring assays, which use RNA counting as a measure of gene expression, were conducted. RNA-based intrinsic subtype was determined using the PAM50 gene signature described by Parker et al.^2^ Based on the highest Pearson correlation with a subtype-defined centroid, each tumor was categorized into one of five intrinsic subtypes (Luminal A, Luminal B, HER2, Basal-like, Normal-like), using the 50 gene, PAM50 signature.^27^ Categorizations were based on a previously validated risk of recurrence score, generated using PAM50 subtype, tumor proliferation, and tumor size (ROR-PT) with a cutoff for high of 64.7 from the continuous ROR-PR score.^2^

### Image analysis pre-processing and feature extraction

Color and intensity normalization was first applied to standardize the appearance across core images, countering effects due to different stain amounts and protocols, as well as slide fading.^29^ The resulting stain intensity channels were then used as input to the rest of our algorithm. Most automated analyses of histology images use features that describe the properties of cells such as statistics of shape and color.^5,30–32^ Such features are focused on cell-by-cell morphology and do not adapt well to new data sets. We instead captured tissue properties with a Convolutional Neural Network (CNN), which has been shown more successful for classification tasks on histology.^16,33^ These multi-layered networks consist of convolution filters applied to small patches of the image, followed by data reduction or pooling layers. Similar to human visual processing, the low level filters detect small structures such as edges and blobs. Intermediate layers capture increasingly complex properties like shape and texture. The top layers of the network are able to represent object parts like faces or bicycle tires. The convolution filters are learned from data, creating discriminating features at multiple levels of abstraction. There is no need to hand craft features. We used the VGG16 architecture (configuration D)^34^ that was pre-trained on the ImageNet data set, which consists of 1.2 million images from 1000 categories of objects and scenes. Although ImageNet contains a vastly different type of image, CNNs trained on this data set have been shown to transfer well to other data sets,^35–37^ including those from biomedical applications.^14,38^ The lower layers of a CNN are fairly generic, while the upper layers are much more specialized. The lower layers only capture smaller-scale features, which do not provide enough discriminating ability, while the upper layers are so specific to ImageNet that they do not generalize well to histology. Intermediate layers are both generalizable and discriminative for other tasks. In transferring to histology, we must search for the layer that transfers best to our task. Output from each set of convolutional layers, before max pooling, was extracted over each image at full resolution to form a set of features for the image. Output from the fourth set of convolutional layers was chosen because it performed better than the outputs from other layers. The fourth set of convolutional layers outputs features of dimension 512. These lower CNN layers are convolutional, meaning that they can be run on any image size. For an image size of 2500 × 2500, they produce a grid of 284 × 284 × 512 features.

### Model training and training data sets

In training a model to predict the class or characteristic group of a tumor, such as high or low grade, we utilize patient-level labels. The TMA images are much larger than the required input to the VGG16 CNN (i.e., typically 2500 × 2500 pixels for TMA spots vs. 224 × 224 for VGG16). Further, applying the original CNN fully convolutionally would produce features that are not generalizable to histology. Thus, some modifications to the VGG16 approach are necessary. A new classifier must be trained to operate on the intermediate level features from VGG16. Simply taking the mean of each feature over the image would limit our insight into which parts of the image contributed to the classification. The patient-level labels are weak compared to detailed patch- or pixel-level annotations used in most prior work, necessitating a different classification framework called multiple instance learning. In this setting, we were given a set of tumors, each containing one or more image regions. We were given a label for each tumor: tumor grade (pathologist determined), ER status (IHC-based), PAM50 intrinsic subtype (50 gene expression-based), ROR-PT (gene expression-based), or histologic subtype (pathologist determined). Due to the diverse appearance of tissue in a single image, learning the model with the patient label applied to every image region did not perform well in initial experiments. Heterogeneity of image region labels in each image is instead accounted for while training the model.

In order to account for intra-tumor heterogeneity, a probabilistic model was formed for how likely each image region is to belong to each class, with these probabilities aggregated across all image regions to form a prediction for the tumor as a whole. Image regions were generated as 800 × 800 pixel regions in the training images, with the mean of each CNN feature computed over the region. A linear support vector machine (SVM)^39^ calibrated with isotonic regression^40^ was used to predict the probability for each region. Isotonic regression fits a piecewise-constant non-decreasing function, transforming the distance from the separating hyperplane learned by the SVM to a probability that an image region belongs to each class. This assumes that the SVM can rank image regions accurately and only needs the distances converted to probabilities. Each image region was labeled with the class of the tumor from which it belongs. The data for model fitting and calibration must be disjoint, so cross-validation was used to split the training instances into five equal-sized groups, where four were used for training and the remaining for calibration/validation (the test set remains untouched). For each fold, an SVM was learned on the training set and calibration was learned on the calibration set with isotonic regression, thus forming an ensemble. An ensemble of size five was selected to balance the desirability of a large training set, a reasonably sized validation set, and the simultaneous desirability of limiting the computation time. Predictions on the test set were made by averaging probabilities from the five models. This ensemble method also helped to soften any noise in the predictions caused by incorrect image region labels due to heterogeneity.

Predictions for tumors were made by first forming a quantile function (inverse cumulative distribution) of the calibrated SVM ensemble predictions for the image regions using 16 equally spaced quantiles from images in the training set. The quantiles of the training images were used to train another linear SVM to predict the class label for the whole tumor, with sigmoid calibration transforming the SVM output into probabilities. This method allowed predictions to be made for individual image regions, while also aggregating to overall tumor predictions.

When training the previously described SVM classifiers, we initially weighted each class, including tumor grade, ER status, and Basal-like vs. non-Basal-like intrinsic subtype, equally. To reduce the leverage of grade in predicting ER status and intrinsic subtype, sample weighting was applied using weights inversely proportional to the number of samples in the group, i.e., low grade class 1, low grade class 2, high grade class 1, and high grade class 2 were each weighted equally, where the classes are the ER status, histologic subtype, or intrinsic subtype.

### Prediction in test sets

At test time, 800 × 800 pixel overlapping regions with a stride of 400 pixels were used as image regions from each TMA spot that is typically 2500 pixels in diameter. Only image regions containing at least 50% tissue within the core image field of view (i.e., 50% tissue, 50% glass) were used. The calibrated SVM ensemble predicted the class of each image region by assigning a probability of belonging to one of two classes (tumor grade 1 or 3, ER + or ER-, Basal-like or non-Basal-like subtype, ductal or lobular histologic subtype, and low-med or high ROR-PT). The probabilities computed on the image regions from all cores were aggregated into a quantile function and the second SVM was used to predict the class for the whole tumor.

### Image-based classification

Cut points were determined for each tumor characteristic based on the achievement of optimal sensitivity, specificity, and accuracy of each core being correctly classified relative to the pathology or biomarker data. To classify tumor grade, image analysis assigned a probability score of being a high-grade vs. low-grade tumor for each image. A cut point of greater than 0.80 was used for high-grade tumors (Fig. 1a). Independently, traditional pathologist scoring methods were used to classify tumors as a combined grade of low, intermediate, or high. Also, two independent pathologists’ classifications of tumor grade for the same tissue sample were assessed to compare the agreement between two pathologists to that observed for image analysis vs. pathologist classification. To classify patients as ER positive based on image analysis, the same principles were used as those described for tumor grade where each core was assigned a probability of ER-positivity. A probability of greater than 0.50 was classified as ER-positive by image analysis. To classify patients as ER positive based on biomarker data, samples had to have 10% or more of nuclei stained positive for ER by immunohistochemistry. For Basal-like vs. non-Basal-like RNA-based subtype, image analysis assigned a probability of each image being Basal-like and a probability cut point of >0.60 was used to classify Basal-like vs. non-Basal-like tumors. These results were compared against the PAM50-based intrinsic subtype classification methods using gene expression described previously.^2^ Similarly, we used image analysis to predict whether a tumor had a high or low-medium risk of recurrence. Image analysis predicted ROR-PT based on a cut point of 0.20 for the probability of each TMA spot being classified as high ROR-PT. Histologic subtype was restricted to ductal and lobular tumors and was based on a cut point of 0.1 for the probability of each TMA spot being classified as lobular.

### Prediction accuracy and associations with clinical characteristics

For core-level comparisons, image region probabilities were calculated of being a high-grade tumor, ER positive, Basal-like subtype, lobular subtype, or high ROR-PT. For each variable, sensitivity, specificity, accuracy and kappa statistics (95% confidence interval [95% CI]) were determined comparing the image analysis classification to tumor grade for the tumor tissue as a whole, IHC-based ER status for each corresponding TMA core (ER positivity is available for each core rather than just for the whole tumor tissue), PAM50 subtype for the tumor tissue as a whole, histologic subtype for the tumor tissue as a whole, and ROR-PT for the tumor tissue as a whole. Accurate classification was defined as identical classification based on histologic image analysis and biomarker data for the same core. To determine whether any clinical characteristics were associated with an inaccurate image-based call for ER status, we estimated odds ratios (ORs) and 95% confidence intervals (95% CI) for the association between patient characteristics and the accuracy of ER status (i.e. concordant with clinical status vs. discordant with clinical status) (Supplemental Table 1). All statistical analyses were done in SAS version 9.4 (SAS Institute, Cary, NC). *p*-values were two-sided with an alpha of 0.05.

### Code availability

Available upon request.

### Data availability

De-identified data, including selected covariates and histological images, are available upon request.

## Electronic supplementary material

Supplemental Table 1

## Electronic supplementary material

**Supplementary information** accompanies the paper on the *npj Breast Cancer* website (10.1038/s41523-018-0079-1).
