# Supplementary material for: Image analysis with deep learning to predict breast cancer grade, ER status, histologic subtype, and intrinsic subtype
Source: NPJ Breast Cancer. 2018 Sep 3;4:30. doi: 10.1038/s41523-018-0079-1 (PMC6120869; doi:10.1038/s41523-018-0079-1)
Supplement: Supplementary file 1 — Supplemental Table 1 [file 41523_2018_79_MOESM1_ESM.docx]

**Supplemental Table 1. Patient and tumor characteristics associated with inaccuracy of predicted ER status from the test set (n=288)**

|  | Inaccurate | Accurate |  | Chi-squared |
| --- | --- | --- | --- | --- |
| Variable | N (%^1^) | N (%^1^) | OR (95% CI) | p-value |
| Age |  |  |  |  |
| ≤50 years | 23 (30.4) | 110 (27.5) | Ref. | 0.72 |
| >50 years | 20 (69.7) | 135 (72.4) | 0.71 (0.37-1.36) |  |
| Race |  |  |  |  |
| White | 25 (84.3) | 125 (77.6) | Ref. | 0.22 |
| Black | 18 (15.7) | 120 (22.4) | 0.75 (0.39-1.45) |  |
| Grade |  |  |  |  |
| Low-Intermediate | 19 (55.4) | 143 (68.7) | Ref. | 0.17 |
| High | 24 (44.6) | 101 (31.3) | 1.79 (0.93-3.44) |  |
| Missing | 0 | 1 |  |  |
| Stage |  |  |  |  |
| I, II | 39 (85.8) | 220 (91.1) | Ref. | 0.49 |
| III, IV | 4 (14.2) | 25 (8.9) | 0.90 (0.30-2.74) |  |
| Node Status |  |  |  |  |
| Negative | 32 (73.8) | 159 (68.2) | Ref. | 0.53 |
| Positive | 11 (26.2) | 86 (31.8) | 0.64 (0.31-1.32) |  |
| Tumor Size |  |  |  |  |
| ≤2cm | 25 (60.0) | 149 (68.6) | Ref. | 0.38 |
| >2cm | 18 (40.0) | 96 (31.4) | 1.12 (0.58-2.16) |  |
| IHC-based ER Status |  |  |  |  |
| Negative | 19 (37.8) | 72 (20.2) | Ref. | 0.07 |
| Positive | 24 (62.2) | 173 (79.8) | 0.53 (0.27-1.02) |  |
| IHC-based Ki67 Status |  |  |  |  |
| <10% | 22 (56.1) | 154 (67.8) | Ref. | 0.24 |
| ≥10% | 21 (43.9) | 91 (32.2) | 1.61 (0.84-3.10) |  |
| Mitotic Grade |  |  |  |  |
| 1 | 14 (40.1) | 116 (58.3) | Ref. | 0.20 |
| 2 | 9 (20.9) | 33 (12.4) | 2.26 (0.90-5.68) |  |
| 3 | 20 (39.0) | 95 (29.3) | 1.74 (0.84-3.64) |  |
| Missing | 0 | 1 |  |  |
| Intrinsic Subtype |  |  |  |  |
| Luminal A | 5 (25.1) | 69 (50.6) | Ref. | 0.41 |
| Luminal B | 8 (26.5) | 25 (20.0) | 4.42 (1.32-14.77) |  |
| Basal-like | 9 (32.4) | 40 (19.8) | 3.10 (0.97-9.91) |  |
| HER2 | 2 (12.4) | 13 (4.8) | 2.12 (0.37-12.14) |  |
| Normal-like | 2 (3.6) | 7 (4.7) | 3.94 (0.64-24.2) |  |
| Missing | 17 | 91 |  |  |

**^1^**All percentages weighted for sampling design
